# The evolution of minimally invasive tissue sampling in postmortem examination: a narrative review

**DOI:** 10.1080/16549716.2020.1792682

**Published:** 2020-07-27

**Authors:** Christina R. Paganelli, Norman J. Goco, Elizabeth M. McClure, Kathryn K. Banke, Dianna M. Blau, Robert F. Breiman, Clara Menéndez, Natalia Rakislova, Quique Bassat

**Affiliations:** a RTI International, Seattle, WA, USA; b RTI International, Research Triangle Park, NC, USA; c Centers for Disease Control and Prevention, Atlanta, GA, USA; d Department of Global Health, Rollins School of Public Health, Emory University, Atlanta, GA, USA; e ISGlobal Barcelona Institute for Global Health, Hospital Clínic – Universitat de Barcelona, Barcelona, Spain; f ISGlobal Department of Pathology Barcelona Institute for Global Health, Hospital Clínic – Universitat de Barcelona, Barcelona, Spain

**Keywords:** Percutaneous autopsy, needle-based autopsy, autopsy, less-invasive autopsy, minimally invasive autopsy

## Abstract

**Background:**

Because of low acceptance rates and limited capacity, complete diagnostic autopsies (CDAs) are seldom conducted in low- and middle-income countries (LMICs). There have been growing investments in less-invasive postmortem examination methodologies, including needle-based autopsy, known as minimally invasive autopsy or minimally invasive tissue sampling (MITS). MITS has been shown to be a feasible and informative alternative to CDA for cause of death investigation and mortality surveillance purposes.

**Objective:**

The aim of this narrative review is to describe historical use and evolution of needle-based postmortem procedures as a tool to ascertain the cause of death, especially in LMICs.

**Methods:**

Key word searches were conducted in PubMed and EBSCO in 2018 and 2019. Abstracts were reviewed against inclusion and exclusion criteria. Full publications were reviewed for those abstracts meeting inclusion criteria and a start set was established. A snowball search methodology was used and references for all publications meeting inclusion criteria were manually reviewed until saturation was reached.

**Results:**

A total of 1,177 publications were initially screened. Following an iterative review of references, 79 publications were included in this review. Twenty-nine studies, published between 1955 and 2019, included MITS as part of postmortem examination. Of the publications included, 76% (60/79) have publication dates after 2010. More than 60% of all publications included addressed MITS in LMICs, and a total of nine publications compared MITS with CDA.

**Conclusions:**

Although there is evidence of less-invasive postmortem sampling starting in the 1800s, more structured needle-based postmortem examination publications started to appear in the mid-twentieth century. Early studies were mostly conducted in high-income countries but starting in 2010 the number of publications began to increase, and a growing number of studies were conducted in LMICs. Initial studies in LMICs were disease-specific but since 2015 have evolved to include more expansive postmortem examination.

## Background

Traditional pathological autopsy, also known as complete diagnostic autopsy (CDA), remains the gold standard methodology to investigate cause of death. However, since the beginning of the mid 1960s, declining rates of CDA have been well documented globally, and this approach to investigate cause of death remains infrequently performed in low- and middle-income countries (LMICs) [1,2]. The reason for the limited use of CDA in LMICs is multifactorial and includes social, cultural, religious, and structural factors such as limited human capacity and financial resources, an overall poor acceptability because of the disfiguring nature of the procedure, and the time required to carry it out, which can interfere with ceremonial and burial practices [3–5]. In high-income countries (HICs), the abundance of clinical records (and their easy accessibility) often allows adequate characterization of events preceding death, and thus, causes of the fatal outcome. However, this is not generally the case in LMICs, where the vast majority of premature deaths occur; in these settings, access to the health system is much more limited, resulting in a significant proportion of deaths occurring in the home. Alternative strategies such as verbal autopsy, a structured interview of the family members that is subsequently analyzed by clinicians or by analytical software, have been designed to ascertain causes of death but provide limited specificity and can give misleading results [6,7]. In these settings, clinical records of those deaths having reached the health system are valuable assets but when available typically have limited information and often entail a significant proportion of diagnostic errors, probably because of the scarcity of diagnostic tools available to clinicians [8]. Thus, the substantial shortcomings of verbal autopsy and clinical diagnosis, especially when not used in conjunction with other sources of information, significantly limit their ability to inform reliable cause-of-mortality data [5,7,9].

Accurate determination of causes of death is critical to guide effective preventive strategies and health systems planning. Because CDAs are not readily conducted in LMICs, alternative methods based on less-invasive postmortem approaches have been historically proposed to investigate cause of death. In this respect, minimally invasive tissue sampling (MITS), also known as minimally invasive autopsy (MIA), has been identified as a possible alternative to CDA to determine cause of death. MITS is a protocolized needle-based postmortem examination, designed as an acceptable proxy of the gold standard CDA, that can be performed by pathologists or pathology technicians with specialized training. MITS consists of inserting fine needles into the body and collecting small amounts of tissue and body fluids (e.g. blood, cerebrospinal fluid, effusions) from key and highly informative organs like the brain, lungs, liver, heart, and placenta (if applicable) [10,11]. These samples are then analyzed through standard and advanced histopathological, microbiological, and molecular biology methods, providing rich information on abnormalities present and likely responsible for the events leading to death.

The concept of minimally invasive postmortem study as a means to support cause of death determination dates back to the late 1800s in Baltimore, Maryland, where Dr. Howard Kelly described removing organs manually for autopsy through body orifices [12]. Some decades later in 1930, Décio Parreiras and Werneck Genofre used needle-based postmortem examination during a yellow fever outbreak in Brazil and hence developed the ‘Parreiras-Genofre Spindle’ used for targeted postmortem liver sampling, also known as ‘viscerotomy’ [13,14]. Over the last decade there have been growing investments in MITS-related studies. These investments have largely but not exclusively targeted the use of MITS for mortality surveillance purposes, particularly in children [15]. To better understand the growth and expansion of needle-based postmortem examination, or MITS, over time, we sought to identify key publications that have contributed to the evolution of MITS as a method to assist physicians and medical professionals in determining cause of death. Specifically, this review addressed the following questions:

- How has postmortem tissue sampling evolved toward the current configuration of MITS to support cause of death investigation in LMICs, and what do we know of its potential acceptability and feasibility?
- How has the MITS technique been validated against the CDA to assess whether it is an acceptable proxy for the gold standard CDA?

## Methods

### Database search and article selection

After an initial July 2018 search in COCHRANE for existing published reviews, a start set was established by searching English key words in PubMed and EBSCO. Non-English databases and publications were excluded. There were no date limitations set on the year of publication. Key words for the search included ‘minimally invasive tissue sampling’, ‘minimally invasive autopsy’, ‘percutaneous autopsy’, ‘needle-based postmortem examination’, and ‘needle-based autopsy’.

The term ‘minimally invasive autopsy’ has historically been used to cover a range of less-invasive autopsy methods including the use of advanced imaging techniques such as magnetic resonance imaging, computerized tomography, and ultrasonography, collectively referred to as ‘virtuopsy’ but which did not always include associated tissue sampling. For this review, we focused on postmortem examinations that were not full pathological autopsies and that were less-invasive or less comprehensive than CDAs and included targeted postmortem tissue sampling. Inclusion criteria consisted of MIA that included needle-based tissue sampling. Search results that included needle-based autopsy in conjunction with advanced imaging techniques were excluded as were publications that included MIA in the absence of needle-based autopsy. See Table 1 for inclusion and exclusion criteria.

**Table 1.** Inclusion and exclusion criteria.

Inclusion Criteria	Exclusion Criteria
Needle-based tissue sampling used in postmortem examination without advanced imaging techniques English language–based publication or database	Use of magnetic resonance imaging, computerized tomography, ultrasonography Non-English database or publication Use of needle-based tissue sampling in live patients Use of needle-based tissue sampling/biopsy for identification of neoplasms (in living or deceased patients)

Because of negative connotations with the word ‘autopsy,’ there was a judgment by researchers investigating the feasibility and acceptability of needle-based postmortem tissue sampling to exclude the word ‘autopsy’ in the procedure terminology because the word could convey an impression about the procedure that would interfere with acceptance. Hence the terminology was changed to MITS. The original language from the reference publications included both ‘MIA’ and ‘MITS’ but for the purposes of this review, the term ‘MITS’ is used to describe all needle-based autopsies, irrespective of whether MIA or MITS was used in the original text. Publications including tissue sampling in live patients were excluded as were publications where tissue sampling was used for the diagnosis of structural defects.

**Figure 1.**
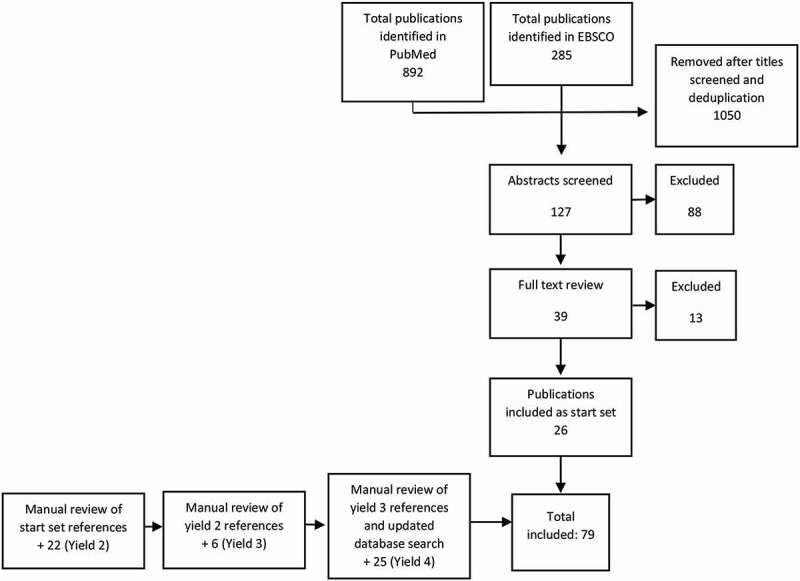
Flow diagram of publication screening and identification.

A snowball methodology was applied to identify publications for this review [16,17]. After an initial search in PubMed and EBSCO, all publication titles were manually reviewed by a single person (CP) against inclusion and exclusion criteria, and duplicates were removed. Abstracts for all publications initially identified were reviewed, and a definitive decision to include or exclude the publication was made after reading the full text, thus establishing a start set. From there, a backward and forward snowball search methodology was applied to establish yields two, three and four (Figure 1). In the backward approach, the titles and authors in the reference lists for all publications included as part of the start set were manually screened, and for those meeting provisional inclusion criteria the abstract was read. After reading the abstracts, the full text of those meeting the inclusion criteria was read and a final determination regarding inclusion was made. In the forward snowball approach PubMed was used to identify citations of publications included as part of the start set. Titles and authors were screened, and abstracts were read for those meeting provisional inclusion criteria. The full text was read before we made a final determination about inclusion. Both backward and forward iterative approaches were conducted until saturation was reached and no new references were identified. In addition to using PubMed alerts, updated searches of PubMed and EBSCO using the same initial search terms were conducted in February 2018 and October 2019 to identify new publications since the original July 2018 search (Figure 1).

**Figure 2.**
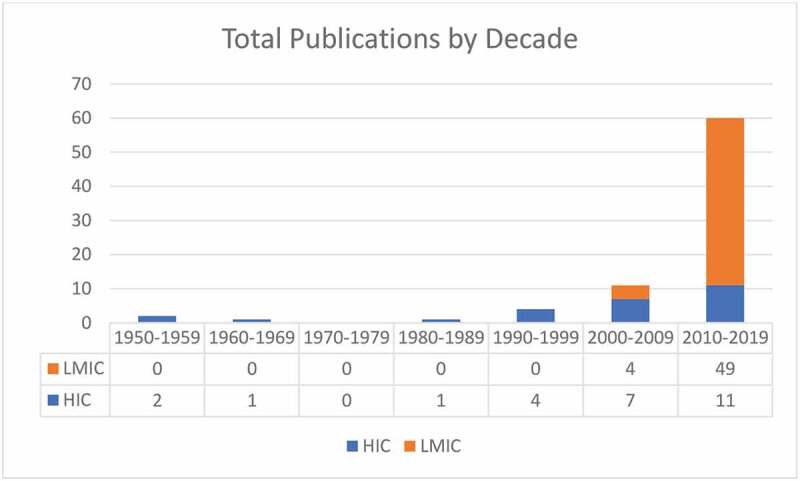
Number of MITS publications by decade focused on HICs and LMICs.

**Figure 3.**
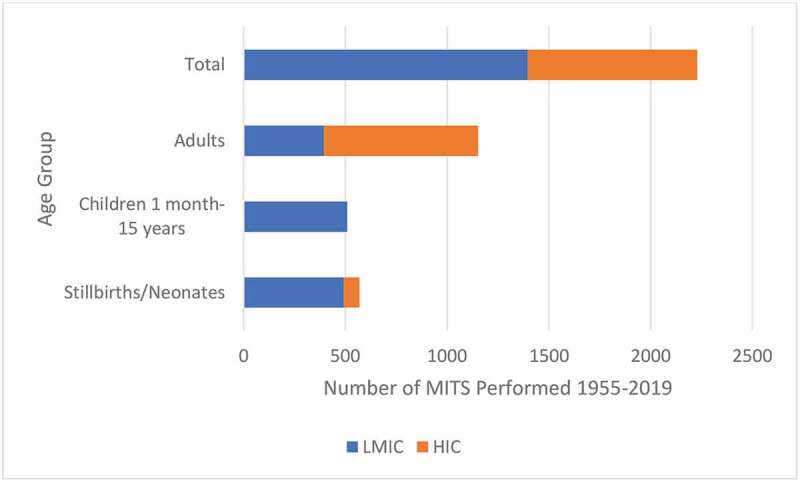
Number of MITS performed in LMICs and HICs by age group, 1955–2019.

### Definitions

We reviewed the country/countries in which each study was implemented and classified them as either HICs or LMICs based on the World Bank Country and Lending Group’s classification at the time of publication [18].

Unless otherwise specified by the study methods, for this review, stillbirth was defined as a death occurring in the period between 28 weeks of gestational age and birth and not showing any sign of life after delivery; neonatal deaths were defined as those occurring among babies born alive but deceased before 28 days of age; child deaths were defined as those occurring between 28 days and <15 years; and adult deaths were defined as those occurring after 15 years of age.

## Results

A total of 79 journal articles with publication dates ranging from 1955 to 2019 were included in this review. The practice of needle-based postmortem examination to inform cause of death was documented in English-language literature as early as 1955. However, despite the concept of MITS being introduced over a half century ago, 75% of the publications identified were published within the last decade. Since 2010, the total number of MITS publications has been three times the number published prior to 2010.

### The evolution of MITS in postmortem examination

Twenty-eight publications with publication dates ranging from 1969 to 2019 represented 19 distinct primary studies where MITS was used as a postmortem examination method to determine cause of death (Table 2) [10,11,19–44]. Three articles were published in the 1950s and 1960s evaluating the potential utility and feasibility of conducting needle autopsies [29,38,39]. The largest study of MITS in adults was conducted over two decades in New York and consisted of histological examination of samples taken by pathology residents from the liver, kidney, lung, and heart [39]. The authors found that the limited practice of the needle autopsy by pathologists in training reduced the efficiency of the needle-based postmortem examination but that with increased exposure and practice, the needle autopsy could serve as a suitable substitute for CDA. Although no publications were identified between the 1960s and 1980s, these early studies introduced the idea of needle-based postmortem examination.

**Table 2.** Publications using MITS to ascertain cause of death.

						Total MITS Cases	Tissues sampled					
First Author	Year	Journal	Region of Study	Income Level	Population		Brain	Heart	Kidney	Liver	Lung	Other
Terry [29]	1955	Journal of Clinical Pathology	North America	HIC	Adults	24				x	x	
West [38]	1957	American Journal of Medical Science	North America	HIC	Adults	66				x	x	Pancreas Prostate
Wellman [39]	1969	American Journal of Clinical Pathology	North America	HIC	Adults	394		x	x	x	x	
Underwood [40]	1983	BMJ	Europe	HIC	Adults	5		x		x	x	Spleen Prostate
Baumgart [41]	1994	Pathology	Europe	HIC	Adults	16		x	x	x	x	Muscle tissue Spleen Stomach Pancreas Bowel Aorta
Foroudi [42]	1995	Pathology	Australia	HIC	Adults	21	x	x	x	x	x	
Huston [43]	1996	Modern Pathology	North America	HIC	Adults	20	x		x	x	x	Breast Bone marrow Abdominal mass
Guerra [44]	2001	Pathology Research and Practice	Europe	HIC	Adults	168	x	x	x	x	x	Spleen Bone marrow
Chintu [19]	2002	Lancet	Africa	LMIC	Children	264		x			x	
El-Reshaid [20]	2005	Medical Principles and Practice	Middle East	HIC	Adults	19		x	x	x	x	
Weustink [21]	2009	Radiology	Europe	HIC	Adults	30		x			x	
Garg [22]	2009	Fetal and Pediatric Pathology	SE Asia	LMIC	Stillbirths/ neonates	25	x			x	x	Spleen
Celiloglu [23]	2013	Turkish Pathology	Europe	HIC	Stillbirths/ neonates	76	x	x	x	x	x	Spleen
Cox [24]	2014	Journal of Acquired Immune Deficiency Syndrome	Africa	LMIC	Adults	191*	x	x	x	x	x	Spleen
Cox [25]	2014	BMC Clinical Pathology	Africa	LMIC	Adults	191*	x	x	x	x	x	Spleen
Castillo [11]	2015	PLoS One	Africa	LMIC	Adults	30 ^Ϯ^	x	x	x	x	x	Blood CSF Bone marrow Uterus
Castillo [27]	2016	PLoS Medicine	Africa	LMIC	Adults	112 ^Ϯ^		x	x	x	x	Spleen CSF Bone marrow Blood
Martínez [11]	2016	Diagnostic Microbiology and Infectious Disease	Africa	LMIC	Adults	30 ^Ϯ^				x	x	CSF Blood Uterus
Karat [26]	2016	PLoS One	Africa	LMIC	Adults	34^§^				x	x	CSF Spleen
Menendez [32]	2017	PLoS Medicine	Africa	LMIC	Stillbirths/ neonates	59	x			x	x	Blood CSF
Bassat Q [31]	2017	PLoS Medicine	Africa	LMIC	Children	54	x	x	x	x	x	Blood CSF Spleen Bone marrow CSF
Castillo [10]	2017	PLoS Medicine	Africa	LMIC	Adults	57 ^Ϯ^	x	x	x	x	x	Blood CSF Spleen Bone marrow Uterus
Karat [28]	2017	PLoS One	Africa	LMIC	Adults	34^§^				x	x	Blood CSF Spleen Naso/oro pharyngeal secretions
Chawana [33]	2019	Clinical Infectious Diseases	Africa	LMIC	Children	127	x			x	x	Blood CSF Rectal swabs
Muhe [35]	2019	Lancet Global Health	Africa	LMIC	Stillbirths/ neonates	126	x		x	x	x	Spleen Intestines
Roberts [34]	2019	American Journal of Clinical Pathology	Africa	LMIC	Children	64					x	
Madhi [36]	2019	Clinical Infectious Diseases	Africa	LMIC	Stillbirths/ neonates	129	x			x	x	Blood CSF Placenta
Madhi [37]	2019	Clinical Infectious Diseases	Africa	LMIC	Stillbirths/ neonates	153	x			x	x	Blood CSF Stool

* Represents same sample population from single study of 191 adults; ^Ϯ^ Represents same sample population from single study of 112 adults; ^§^ Represents same sample population from single study of 34 adults.

In 1983, the use of MITS in HICs resurfaced in a paper describing the advantages, feasibility, and limitations of needle-based postmortem examination [40]. This publication described the use of MITS in five case studies, and the authors concluded that two clear advantages of MITS were the speed at which samples could be obtained and the reduced risk of infection. The next study involving MITS was not published until more than 10 years later when in 1994 the first study demonstrating the use of MITS in HIV-positive populations was published [41]. Two additional studies were published in the mid1990s; one study suggested that MITS is a valuable alternative when CDA is not possible, and the second publication stated that when used in isolation, MITS is inferior, and suggested the use of radiology to improve its performance [42,43].

The early 2000s saw mixed opinions about the feasibility and acceptability of MITS in postmortem examination in the English-language literature in both HICs and LMICs. One study evaluated the feasibility of MITS in a predominantly Muslim culture and found it to be a more acceptable alternative to CDA [20]. However, another study in Zambia evaluating the acceptability of MITS in children found that offering MITS as a less-invasive alternative to CDA did not significantly increase consent [19]. Studies of MITS in children, neonates, and stillbirths conducted in the early 2000s consisted of comparing CDA with MITS and using MITS to confirm a specific condition, for instance, malaria (targeting the brain) or pneumonia (targeting the lungs) [19,22,45]. These studies not only expanded the use of MITS, they introduced new methods for obtaining tissue samples.

Between 2010 and 2015 an increasing number of studies aimed to validate the use of MITS against other methods of postmortem examination, including CDA and verbal autopsy [11,23–25]. During the same timeframe these studies were accompanied by other publications, including articles describing qualitative studies that examined the potential acceptability of MITS as part of postmortem examination, particularly in populations where CDA is rarely performed. Journal commentaries and editorials outlining the perceived utility and value of MITS in postmortem examinations and cause of death determination also began to emerge in the literature [7,46–51].

In 2016 publications began to arise from the pioneering CADMIA study. CADMIA assessed the acceptability of MITS and validated the MITS approach against the CDA in all age groups, including stillbirths in maternal deaths, in Mozambique and in Brazil [27,30–32,52,53]. The year 2019 saw a sharp rise in the quantity of MITS publications, including a study of children dying of respiratory illness in Kenya and a study of stillbirths and neonates in Ethiopia [34,35]. A large proportion of the 2019 increase in MITS publications is attributable to the October 2019 release of 13 articles describing MITS from the experience of the Child Health and Mortality Prevention Surveillance (CHAMPS) Network [2,15,33,36,37,54–62]. With promising results from the relatively few validation studies completed, the CHAMPS Network rapidly endorsed the use of MITS and is poised to both build on earlier validation studies and also improve on a number of aspects of MITS such as reducing the time and expense associated with performing MITS.

### MITS in LMICs and HICs

In 28 publications documenting a total of 19 studies of MITS in postmortem examination and determination of cause of death published between 1955 and 2019, roughly half of the studies were conducted in HICs and half in LMICs. In early MITS studies, between 1955 and 2001, the scope of MITS was limited to HICs and adult populations [29,38–42,44]. Articles published between 1955 and 2010 continued to focus on HICs at a ratio of 4:1 compared to those published about LMICs (Figure 2). The first MITS study conducted in an LMIC was published in 2002 and notably was also the first study of MITS in children [19]. Although two additional studies of MITS in adults in HICs were published in 2005 and 2009, beginning in 2010 there was a significant shift from HICs to LMICs [20,21]. In 2011 the number of publications documenting MITS in postmortem examination in LMICs began to increase, and between 2011 and 2019 articles from LMICs outnumbered those from HICs by a ratio of 3:1 [11,24–28,30–32,34–37].

The number of qualitative studies aimed at understanding the facilitators and barriers to implementing MITS in a variety of cultures, religions, and populations, including health care providers, parents, families, and community leaders is relatively balanced in HICs versus LMICs. Of the seven qualitative studies with publication dates between 2011 and 2019, four were conducted in LMICs and three in HICs [3,49,50,52,58,59,63–65].

Based on the sample sizes and ages identified in the 28 primary studies using MITS in postmortem examination there have been roughly 40% more MITS carried out in LMICs than in HICs. There have been nearly twice as many MITS in adults as in stillbirths, neonates, and children combined; however, in LMICs, the majority of MITS conducted have been in children, neonates, and stillbirths (Figure 3) [10,17–33,35-42,64].

### MITS in specific disease investigations

A total of eight studies published between 1994 and 2019 document the use of MITS to investigate specific diseases or pathogens and to support determining cause of death in cases with multiple comorbidities or when clinical symptoms make it difficult to distinguish between potential causes of death [19,24–26,28,34,41,44,45,67–69].

The use of MITS in determining the role of specific diseases and pathogens in postmortem examination dates back to 1994 and 2001 when it was used in two studies to determine cause of death in HIV-positive adults in Europe [41,44]. Both studies highlighted the limitation of ascertaining cause of death through clinical diagnosis and in particular in HIV-positive adults who were likely to have multiple comorbidities [41,44]. Authors from both studies concluded that MITS is comparable with CDA in cause of death determination, may be invaluable in populations with limited resources, and may present fewer opportunities than CDA to infect technicians and pathologists in populations with high rates of infectious disease. During 2015 to 2017 researchers evaluated MITS against verbal autopsy in determining cause of death from HIV-associated tuberculosis in South Africa [28]. Researchers concluded that when used alone, verbal autopsy underestimated HIV-related mortality in patients with tuberculosis. In Malawi, MITS was found to be an effective technique for sampling brain tissue to confirm cerebral malaria in children who died with a clinical diagnosis of malaria [45]. In this study brain tissue was successfully sampled via a supraorbital approach, a novel approach, and was met with an apparent acceptability of the procedure [68].

### MITS validation compared with CDA

A total of 13 articles published between 1957 and 2019 representing nine unique studies validating the use of MITS against CDA in determining cause of death were included in this review (Table 3).

**Table 3.** Publications comparing MITS with CDA.

First Author	Year	Journal	Specific Study Objective	Population Studied	Total MITS Conducted	Results
West [38]	1957	American Journal of Medical Science	Compare diagnosis made by MITS to CDA	Adults	66	77% of MITS cases were able to reach a diagnosis In 48% of the cases there was agreement between MITS and CDA
Foroudi [42]	1995	Pathology	Compare results of MITS with CDA	Adults	21	Cause of death ascertained in 43% of MITS cases compared to 95% of CDA
Huston [43]	1996	Modern Pathology	Demonstrate the value and limitations of postmortem needle sampling in correlating tissue diagnosis compared with CDA	Adults	20	Cause of death was confirmed by MITS in 60% of the patients MITS confirmed additional diagnosis identified by CDA in 75% of the cases
Cina [67]	1999	Military Medicine	Evaluate the role of percutaneous core biopsy of the liver in determining pathology in unexpected deaths related to hepatomegaly	Adults	28	86% correlation between MITS and CDA
Breeze [73]	2008	Virchows Archive	Determine the feasibility of percutaneous fetal organ biopsies: how frequently are adequate samples obtained; comparison of percutaneous biopsy with conventional block biopsy	Stillbirths	30	46% of samples collected via MITS were considered adequate Adequate samples were obtained from lungs and liver
Garg [22]	2009	Fetal and Pediatric Pathology	Compare the specimen adequacy and histologic and laboratory findings on lung tissue collected using blind-needle biopsy MITS with those from CDA in a pediatric respiratory disease mortality study undertaken at a large public hospital in Kenya, assessing the contribution of molecular microbiologic testing	Neonates	25	Full agreement between CDA and MITS in 56% of cases Partial agreement between CDA and MITS in 12% of cases
Van der Linden [74]	2014	PLoS One	Compare the RNA quality of samples obtained via MITS to samples obtained via CDA with fresh frozen samples as a reference	Adults	24	Samples obtained via MITS had higher quality RNA compared with those collected via CDA
Castillo [27]	2016	PLoS Medicine	Compare cause of death in adults ascertained via MITS with cause of death after CDA	Adults	112	75.9% agreement in MITS and CDA in cause of death determination Able to ascertain cause of death in 89% of MITS cases and 100% of CDA cases
Castillo [10]	2017	PLoS Medicine	Compare cause of death in maternal cases ascertained via MITS with cause of death after CDA	Adults	57	68% agreement between MITS and CDA in cause of death Able to ascertain cause of death in 84% of MITS cases compared with 98% of CDA
Bassat [31]	2017	PLoS Medicine	Compare cause of death in children ascertained via MITS with cause of death after CDA	Children	54	89% concordance in cause of death between MITS and CDA
Menendez [32]	2017	PLoS Medicine	Compare cause of death in stillbirths ascertained via MITS with cause of death after CDA	Stillbirths and neonates	59	68% agreement between CDA and MITS Cause of death ascertained in 83% of MITS cases compared with 89% of CDA cases
Fernandes [75]	2019	Human Pathology	Determine the degree to which clinical information improves the diagnostic accuracy of the CDA and MIA	Adults	112	Cause of death was ascertained in 89% of MITS cases (all populations) that included clinical information compared with 80% of MITS cases without clinical data
				Maternal deaths	57	
				Children	54	
				Neonates	41	
				Stillbirths	18	

The first study we identified comparing MITS and CDA was published in 1957 in North America [38]. The investigators found that in a study of 50 adult patients undergoing first MITS and then CDA, where the pathologists conducting MITS were blinded to the CDA results and clinical histories, the concordance in diagnosis between CDA and MITS was 48%, leaving the authors to conclude that CDA is preferable to MITS [38]. The next article comparing MITS and CDA was not published until 30 years later, in 1995, in Australia where 95% of CDA resulted in a cause of death determination compared with 43% of MITS cases [42]. The authors concluded that MITS was inferior to CDA, but the performance of MITS in determining cause of death might be improved using radiology. The 1990s saw two additional studies comparing MITS and CDA in adults. The first, published in 1996, found a correlation of 67% between MITS and CDA in cause of death determination and 80% correlation between MITS and CDA in additional major diagnoses in adults [43]. The authors concluded that although MITS should not replace CDA when CDA is feasible, MITS can be an efficient and satisfactory alternative, because it can be conducted in a relatively short period of time and in the hospital room prior to transferring to the morgue or funeral home [43]. The second, a study of adults published in 1999, found an 86% correlation in cause of death based on histological findings when comparing liver samples collected via MITS with those collected via CDA [67]. Furthermore, a study of HIV-positive adults in Uganda in 2014 found concordance rates between MITS and CDA in major diagnosis reaching 90%, leaving the authors to conclude that MITS is a viable alternative when CDA is not possible [24]. These studies highlight many of the advantages of MITS; MITS can be conducted in less time and more efficiently than CDA, may be a suitable alternative to families refusing CDA, and may be a safer alternative to CDA in cases of infectious pathogens.

Beginning in 2016, the CADMIA study in Mozambique and the Brazilian Amazon sampled the brain, heart, liver, spleen, kidneys, and lungs and found a high concordance rate in major diagnosis between CDA and MITS and like earlier studies, found that over time sampling technique improved and that where CDA is not possible, MITS is a valuable and robust alternative [10,11,27,30-32,70]. Concordance rates between CDA and MITS ranged from 68% in stillbirths to 89% in pediatric deaths with the highest concordance in deaths attributable to infectious diseases and malignant tumors [31,32]. Among other observations, the authors described that the lungs, liver, and brain tissue sampled using MITS have the greatest diagnostic yield, and the inclusion of clinical data in assigning a cause of death significantly improves the diagnostic capacity of MITS against CDA [8,11]. The CADMIA studies were also responsible for standardizing the MITS protocols for sample collection and histological processing and training of more than 60 project staff for the CHAMPS Network in addition to other MITS projects [11,56,71]. Most recently, in publications from 2019, researchers reported that 60% concordance in pathogen detection between MITS and CDA was observed in the MITS study of specimen adequacy and histologic and laboratory findings in 64 children in Kenya [34]. In Ethiopia, CDA and MITS were used in a mortality study of 125 stillbirth and neonatal deaths [35]. However, results describing the performance of MITS compared with CDA have not yet been published.

## Discussion

This review documented that there were limited publications on the validation and use of MITS in disease-specific cause of death investigations in both HICs and LMICs between 1955 and 2010. MITS was initially studied more in HICs and was not introduced in LMICs until the early 2000s and was almost exclusively used in the context of disease-specific studies. However, 2010 marks the beginning of an increase in the total number MITS publications in both HICs and LMICs. The year 2010 also marks a distinct shift in publications predominantly describing MITS in HICs to an increasing number of publications describing MITS in LMICs. Since 2010, the studies using MITS in LMICs have also expanded the earlier scope from use in disease-specific contexts to include studies evaluating MITS to CDA and more broadly, using MITS in mortality surveillance.

However, it is worth noting that despite the rapid increase in MITS publications in the last decade, the total number of publications cannot, in this case, serve as a proxy for the number of MITS studies, and there remains relatively few studies evaluating MITS against CDA in LMICs. Since 2009, 19 MITS cause of death publications originated from eight unique projects. Seventy-five percent of those projects were funded by a single donor, the Bill & Melinda Gates Foundation. The significance of this data point is two-fold. First, the large investment in MITS suggests a high level of confidence and a deep belief in the value MITS can contribute to improved global mortality surveillance. Second, there remains significant opportunity to broaden the funding sources that support MITS research in mortality surveillance.

One initiative to further stimulate and facilitate the use of MITS to characterize causes of death is the MITS Surveillance Alliance, whose objective is to grow a network of researchers to support the use of MITS globally. To this end, the Alliance recently funded a number of small grants to support MITS feasibility studies in LMICs and the innovative use of MITS in other disease- and pathogen-specific mortality surveillance studies [72]. Such grants highlight both the global interest for MITS and the potential for MITS to refine disease-specific mortality across a broad range of pathogens.

As investments in MITS increase, so do the opportunities to expand and improve the utility of MITS and postmortem examination. For example, future investments in MITS that include funding to study the differences in MITS acceptability based on local customs and cultures and support community engagement will guide researchers in the design and implementation of future MITS studies in LMICs. Results from these qualitative studies will add to the body of literature on the utility of MITS in settings where barriers may prevent adoption and ultimately optimize its acceptance. The prospects of using artificial intelligence and machine learning to systematize and streamline MITS data analysis and interpretation, at both the individual and population level, has yet to be explored. Economic studies aimed at determining the relative cost-effectiveness and cost comparison between MITS and CDA will provide potential funders, researchers, and governments, particularly in LMICs, with valuable information when considering the feasibility of MITS in their respective settings. The use of MITS has been documented in HIV and tuberculosis, but there is still potential for additional targeted use of MITS in other pathogens and infectious disease-specific investigations. Further investment in MITS is needed to support its continued evolution and its role in postmortem examination.

In summary, although the concept of MITS and tissue-based postmortem examination dates back many decades, and the concept of MIA has been around since before the turn of the twentieth century, this review demonstrates a rapid increase in the number of MITS studies in the last decade. The results of this review support the conclusion that MITS is a feasible and comparable alternative method for postmortem examination compared with CDA. However, the literature also suggests that the potential value and impact of MITS in cause of death determination is still relatively unfulfilled. Continued investment, and importantly, multisectoral engagement, have the potential to accelerate MITS validation and adoption globally.

## Paper context

Despite recent increases in the number of minimally invasive tissue sampling publications, this manuscript is the first to comprehensively document individual studies using needle-based postmortem examination in a single publication. This manuscript synthesizes these studies and gives readers deeper insight and a broader perspective into how and where minimally invasive tissue sampling has been performed, the different contexts in which it has been used, and how it has been validated against the complete diagnostic autopsy.
